# Reducing Injury Risk and Improving Skill: How a Psycho-Perceptual-Motor Approach Can Benefit High-Performance Sport

**DOI:** 10.1177/19417381231156437

**Published:** 2023-04-24

**Authors:** Sean Müller, Tim Gabbett, Dominic McNeil

Managing the physical workload of athletes is crucial to guard against potential injury during practice and allow seamless participation in competition.^22^ In the past decade, there has been discussion in the media by former or current professional athletes and coaches, as well as sports journalists, regarding whether physical workload regulations may stifle the skill practice needed to prepare athletes adequately for competition.^7,24^ For example, professional cricket has changed rapidly in the last 10 years, with players participating in 3 different formats (ie, 5-day test matches, 50-over 1-day matches, and 20-20 matches) in shorter touring schedules. Similarly, there can be up to 162 games played in major league baseball, not including spring training. Consequently, workloads are regulated tightly to minimize increases in injury risk that can occur in response to rapid or progressive increases in the number of overs bowled or pitches thrown.^7,18^ Alternatively, in the highly dynamic and physical sport of Australian football, players are required to kick from varying distances to score a goal. Practice of this crucial skill of set-shot kicking has been argued to be limited due to concerns over workload and potential injury.^24^ Yet, there has been a decline in proficiency of skills such as in set-shot kicking,^2^ which may be caused by tightly regulated physical workload limiting skill practice opportunities. This is a major concern as decreased skill proficiency can result in losses for a team and lack of revenue for athletes and coaches.^2,24^ This guest editorial aims to inform high-performance staff within professional sport organizations of psycho-perceptual-motor skill methods, which can facilitate a better balance between physical workload and skill development of athletes.

## Psycho-Perceptual-Motor Skill Approach

Professional athletes and coaches believe that psycho-perceptual-motor skills are crucial for success in elite sport.^9,21^ Psycho-perceptual-motor skills refer to mental and motor skill methods that can be implemented effectively without high physical workload to remediate or accelerate performance.^20,28^ The ‘psycho’ component refers to psychological skills such as regulation of emotions due to anxiety or preperformance routines based upon imagery.^28^ It is well established that psychological skills can enhance performance in sport.^12,23^ It has also been reported that psychological factors such as anxiety during preseason may contribute to increased injury risk of athletes during a season.^16^ The ‘perceptual-motor’ component incorporates cognition referring to use of visual (eg, opponent postural cues) and contextual (eg, opponent action tendencies, game score, positioning of opposition) information for action by a performer such as a cricket or baseball batter or field-hockey goalkeeper.^21^ It is well established that perceptual-motor skill relating to pick-up of visual and contextual information is crucial to performance in sport.^6,20,26^ In addition, the perceptual-motor component refers to practice, feedback and instruction methods designed to represent the target sport that can enhance performance.^1^ It is also well established that specific practice and instruction methods can enhance skill performance.^5,14^ Therefore, incorporation of a psycho-perceptual-motor approach into day-to-day schedules can optimize athlete skill preparation for competition without increased risk of injury or burnout.

### Psychological Component

Psychological skills training should be implemented in consultation with an accredited sport psychologist.^19^ A recent systematic review reported that psychological skills training such as imagery, goal-setting, and self-talk may contribute to reduce risk of injury.^11^ For example, imagery is a psychological skill that involves the rehearsal of components related to a motor skill, which can be done in a variety of locations. Imagery is advantageous as athletes are able to imagine various scenarios experienced during sport, yet imagery rehearsal can be implemented while seated in a quiet room during practice or immediately before competition.^12^ As imagery is overt where the rehearsal occurs mentally, no physical movement is performed, and, in turn, the skill can be developed without increasing the physical workload. Research has reported that imagery conducted on its own can improve decision-making time but, when completed in combination with skill practice, results in superior performance accuracy than skill practice alone.^12,19^ Moreover, imagery can be designed to not only improve execution and tactical aspects of motor skills but also improve psychological factors such as confidence, motivation, and reduction of stress related to performance.^12^ Therefore, practitioners should plan skill practice sessions that incorporate imagery and/or other psychological skills to manage overall physical workload and maximize improvement in motor skill performance.

Bouts of psychological skills training can be incorporated to balance physical load and skill practice across training sessions. For example, in team sports such as cricket or baseball, batters and bowlers or pitchers could engage in routine net or simulated game practice for approximately 40 minutes, then be rotated to a quiet room to complete approximately 20 minutes of imagery training. Thereafter, they could return to routine net or simulated game practice for the remainder of its duration. Such incorporation of psychological skills training into routine professional sport skill practice has been reported to improve performance and has been welcomed by professional athletes as better preparation for competition than traditional practice.^3,27^

### Perceptual-Motor Component

Perceptual-cognitive-motor skill training should be implemented in consultation with a motor skill learning specialist.^21^ Typically, psycho-perceptual-motor methods are implemented to accelerate performance taking into consideration individual athlete capabilities and deficiencies, as well as cognitive load and task difficulty relative to closed (self-paced) and open (externally paced) skills.^13^ It is commonly believed though that high volumes of repetitive practice of specific technique is consistently needed to develop high skill proficiency.^8^ This suggests that increased physical workload is needed to develop high level skill. However, high volumes of repetitive specific skill practice is associated with increased risk of injury in players.^15,17^ To guard against risk of injury from repetitive practice physical load, variable practice can be employed. Research indicates that repetitive blocked practice of a consistent technique and skill goal results in superior performance during the practice session, but variable practice involving intricate alterations in technical execution and skill goals results in superior skill performance at a later instance in time.^5,14^ Furthermore, research indicates that performance can be enhanced when video simulation training incorporating pick-up of contextual and opponent postural information is combined with routine skill practice, compared with skill practice conducted alone.^20^ Simulation training can be implemented through video or virtual reality with a simulated action or no action to minimize physical workload.^10,25,29^ Therefore, practitioners should plan variable technical practice and simulation training within skill practice sessions.

Like psychological skills training, bouts of perceptual-cognitive-motor skill training can be easily incorporated into routine practice to manage physical load and skill practice. Using the cricket and baseball example from above, in a net session, bowlers or pitchers can be required to bowl or throw different ball types every 3 trials in a variable practice schedule. This will require intricate variations in technique and muscles used to achieve subtle variations in skill outcome and accuracy. In a different session, the focus could be upon simulation training and/or imagery. For example, after a proportion of routine practice time in the nets, batters can be rotated to complete a bout of video simulation training lasting around 20 minutes in a room. Simultaneously, bowlers or pitchers can be rotated to complete an approximately 20-minute bout of imagery in a quiet room. Such incorporation of visual simulation training within routine practice has been reported to improve performance in professional athletes.^4,20^

## Conclusion

The key message is that preparation of athletes’ skill for competition can be achieved through methods that do not require increased physical workload and, in turn, potential for increased injury risk. This does not mean that routine physical skill practice is less of a priority for skill improvement. Rather, a psycho-perceptual-motor approach should be combined with routine practice to maximize performance without drastically increasing the potential risk of injury to the athlete. A rich opportunity exists for high-performance departments of sports organizations to implement psycho-perceptual-motor methods to maximize athlete preparation time for competition.


—Sean Müller, PhD *Centre for Smart Analytics, Federation University,
Ballarat, Victoria, Australia*
—Tim Gabbett, PhD *Health Innovation and Transformation Centre, Federation
University, Ballarat, Victoria, Australia; Gabbett Performance Solutions, Brisbane,
Australia*
—Dominic McNeil, PhD *Institute of Health and Wellbeing, Federation
University, Ballarat, Victoria, Australia*

